# Mosses as extraordinary reservoir of microbial diversity: a comparative analysing of co-occurring ‘plant-moss twins’ in natural alpine ecosystem

**DOI:** 10.1186/s40793-025-00728-z

**Published:** 2025-06-04

**Authors:** Dinesh Kumar Ramakrishnan, Birgit Wassermann, Christian Berg, Ahmed Abdelfattah, Gabriele Berg

**Affiliations:** 1 Leibniz Institute for Agricultural Engineering and Bioeconomy (ATB), Max-Eyth-Allee 100, 14469 Potsdam, Potsdam, Germany; 2 Institute for Biochemistry and Biology, University of Potsdam, Karl-Liebknecht-Str. 24/25, 14476 Potsdam, Germany; 3 Institute of Environmental Biotechnology, Graz University of Technology, Petersgasse 12, Graz, 8010 Austria; 4 Institute of Biology, Department of Plant Sciences, NAWI Graz, University of Graz, Graz, 8010 Austria

**Keywords:** Alpine ecosystem, Moss and vascular plant microbiomes, Soil type and microbial diversity, Phyllosphere

## Abstract

The decline in plant biodiversity is evident at global scale, but little is known about the loss of microbial diversity associated with diverse plant phyla and their influencing factors. This study investigates the microbial diversity associated with mosses and co-occurring vascular plants in the Alpine ecosystem, focusing on 52 plant “twins” growing on contrasting soil types (carbonate and silicate). Despite co-occurring in the same soil, mosses harbored significantly higher microbial richness and diversity than vascular plants. Across all samples, mosses supporting a total of 3,435 bacterial ASVs and 1,174 fungal ASVs. In contrast, vascular plants hosted a total of 1,760 bacterial ASVs and 911 fungal ASVs. Plant phyla strongly influenced microbial community composition, with vascular plants exhibiting a selective microbial assembly strategy, while mosses showed greater environmental influence. Soil type significantly influenced microbial composition in both plant types, with carbonate soils supporting greater bacterial richness, particularly in mosses. Linear discriminant analysis effect size (LEfSe) analysis highlighted consistent enrichments of *Proteobacteria*, i.e., *Sphingomonadales*,* Rhizobiales*,* Burkholderiales* and, *Pseudomonadales*, in vascular plants across soil types, whereas mosses displayed distinct microbial enrichment patterns between carbonate and silicate soils, suggesting a higher environmental connectedness. Our findings demonstrated that plant phyla are a major determinant of the phyllosphere microbiota, and that mosses represent a currently untapped source of microbial biodiversity. This study highlights the importance of considering both host traits and environmental factors for protecting microbial biodiversity and implementing them in global strategies for restoring biodiversity.

**Supplementary Information:**

The online version contains supplementary material available at 10.1186/s40793-025-00728-z.

## Introduction

The footprint of the Anthropocene is reflected in the planetary boundary concept [1], with biodiversity loss standing out as a critical issue (www.ipbes.net). Recent findings from the largest global plant survey reveal alarming extinction rates for native plants– up to 500 times higher than expected under natural conditions [2]. The extinction of these plants could lead to the loss of their associated microbes, disrupting ecosystem functions and reducing resilience [3–6]. Recognizing this correlation represents a breakthrough in understanding the significance of microbial biodiversity, yet it necessitates a deeper exploration of underlying drivers, mechanisms, and potential solutions [7, 8]. These host-specific microbiomes can significantly influence plant traits, highlighting the functional importance of microbial communities in plant adaptation and evolution [9].

For over two decades, it has been well established that plants are associated with host-specific microbiomes, influenced by both host identity and environmental conditions [10]. Mosses and vascular plants, two distinct lineages of land plants, have evolved unique morphologies, physiologies, and ecological niches that significantly influence their interactions with the surrounding environment, including their associated microbiomes [11]. Vascular plants, with their extensive root systems and diverse physiological processes, harbor complex and dynamic microbial communities that are largely shaped by root exudates and other plant-derived compounds [12, 13]. Studies have demonstrated that different vascular plant species harbor distinct microbial communities, even when grown in the same soil [14–16]. Although, mosses are less studied than vascular plants and despite their simpler structures and lack of true roots [17], different moss species have been shown to harbor distinct bacterial communities, independent of geographical location [18–21]. Furthermore, a recent study has shown a strong phylogenetic coherence of mosses and their associated bacterial communities, indicating a potential evolutionary influence on microbiome assembly [22].

Soil type is another critical factor influencing microbial assembly. Calcareous soils, formed from carbonate rich rocks like limestone and dolomite, are typically alkaline (pH 7–8), porous, and high in plant available calcium but deficient in nutrients such as phosphate, iron, and potassium. In contrast, siliceous soils, derived from silicate rich rocks like granite or gneiss, are more acidic (pH 4–5), less permeable, and tend to accumulate exchangeable aluminium while being depleted in calcium and magnesium. These contrasting physicochemical properties influence not only plant communities but also microbial composition through the differences in nutrient availability and elemental stress [23, 24]. Alpine ecosystems are renowned for the ‘calcareous riddle,’ characterized by the high diversity of calciphilous species [25]. Soil type, through its influence on soil microbial communities, indirectly shapes the microbiomes of aerial plant compartments, including leaves and flowers. Recent findings indicate that soil type (carbonate vs. silicate) shapes microbial community composition but does not significantly affect microbial diversity in aerial plant parts [26]. Therefore, host identity plays a crucial role in shaping plant microbiomes, whereas the soil environment itself acts as both a reservoir of microbial diversity and a selective force in microbiome assembly. As Fierer & Jackson (2006) demonstrated in a continental-scale study, different soil types harbor distinct microbial communities, with edaphic factors, particularly pH, playing a dominant role in shaping microbial biogeography [27]. Since soil is the main source of the plant microbiome, changes in soil microbial communities have a significant effect on plant microbiome assembly. This effect is seen not only in vascular plants but also in mosses. For example, Tveit et al. (2020) reported that microbial communities in peatlands differ significantly between *Sphagnum*- and *Amblystegiaceae-*dominated habitats, likely due to variations in soil properties associated with each peatland type [28]. Moreover, Bragina et al. (2015) showed that diverse plant species, including both bryophytes and vascular plants, share a significant portion of their microbiome in bog ecosystems [29].

The study was conducted as part of the 6th GEO Day of Nature 2021 [30] to investigate the microbial diversity associated with native alpine plants. The study area, located in the UNESCO Biosphere Reserve Salzburger Lungau and Carinthian Nockberge Mountains (“Am Fuß der Zunderwand”), is a biodiversity hotspot where 349 different species of vascular plants and 135 different bryophyte species were documented [30]. At this site, we designed a study that compared the phyllosphere microbiomes of co-occurring moss and vascular plant “twins” growing at the same point with a maximum distance of 50 centimetres within an area of 4 square kilometres. We sampled 52 vascular plant species from 24 families and 52 moss species from 18 families. This is one of the first studies to directly compare these communities under the combined influence of plant type, and soil type and their interaction. Specifically, we aimed to (1) compare the diversity and composition of bacterial and fungal communities in the mosses and vascular plants; (2) determine the relative influence of plant type (moss vs. vascular plant) and soil type (carbonate vs. silicate) on microbiota assembly; and (3) identify specific bacterial and fungal taxa that are differentially enriched in association with each plant type and soil type combination.

## Materials and methods

### Study area and sampling

The study was conducted in the Biosphärenpark Nockberge Mountain, Austria. The Nockberge Biosphere Reserve is protected by the UNESCO MAB (Man and the Biosphere) Programme, and provides a representative example of inner-alpine landscapes with high mountains and deep valleys ranging from 600 m to 3,000 m above sea level with great biodiversity (https://www.unesco.org/en/mab). To compare the phyllosphere microbial diversity and community composition associated with mosses vs. vascular plants, the sampling was designed to collect pairs of co-occurring moss and vascular plant individuals growing at the same point with a maximum distance of 50 cm between them. These moss and vascular plant “twins” represent typical, abundant and rare plant species of the local plant communities, which differ according to soil characteristics and altitude [30]. Our intention behind this was to record as wide a variety of plants as possible in order to obtain a comprehensive picture of plant-associated microbial diversity. A total of 104 samples were collected from 52 randomly chosen sampling points within an area of 4 square kilometres across Biosphärenpark Nockberge (coordinates: 46°52’01.6"N ∼ 46°52’42.9"N; 13°44’01.6"E ∼ 13°43’51.5"E). These samples comprised 52 distinct vascular plant species representing 24 families, and 52 distinct moss species representing 18 families. Detailed information on sampling coordinates and habitat type for each location were recorded (Supplementary Table 1). We did not collect soil samples in this study. Instead, we classified soil types based on site specific geological and vegetation metadata. A subset of sampling sites were classified as either carbonate or silicate based on habitat types identified from geological maps and previous botanical surveys [30]. Specifically, silicate sites included habitats such as thin soil over silicate rock, dwarf shrub heath, and wet nardetum, while carbonate sites were categorized by calcareous rocky grassland or moist calcareous rock fissure. However, we acknowledge that the inclusion of soil samples in future studies would strengthen the understanding of these relationship between soil and plant microbiomes. From each plant individual, approximately 1 g of above ground biomass (leaf tissue for vascular plants, and whole gametophyte for mosses) was collected, depending on moisture levels and sample availability. The specimens were kept at 4 °C during transport to the laboratory and stored at -70 °C. Samples were lyophilized for 48 h using the Labconco Freeze Dry System (Labconco, Kansas City, USA). After lyophilization, the samples were stored at -20 °C until DNA extraction.

### Microbial DNA extraction and amplicon library construction

Samples of moss and plants were ground under sterile conditions with liquid nitrogen, and the total DNA was extracted using the FastDNA Spin Kit for Soil (MP Biomedicals, Solon, USA) and a FastPrep Instrument (MP Biomedicals, Illkirch, France) for 30 s at 5.0 ms^− 1^. Illumina amplicon sequencing was performed by using two different barcoded primer combinations: 515F (5’-GTGCCAGCMGCCGCGGTAA-3’) and 806R (5’-GGACTACHVGGGTWTCTAAT-3’) to amplify 16 S rRNA gene V4 region [31, 32], following the Earth Microbiome Project (EMP) protocol [33]. These primers included adapters for Illumina library preparation and sequencing. One microliter (µL) of extracted DNA was used in each 30-µL reaction. The reaction mixture contained 6 µL (5x Taq&GO, MP Biomedicals), 0.6 µL (10 µM 515 F/806R) primers, 0.45 µL (50 µM mPNA and pPNA), and 20.9 µL of PCR grade water. The peptide nucleic acid (PNA) PCR clamps were used to block the amplification of plastid and mitochondrial 16 S rRNA gene of plants during the PCR amplification of bacterial community [34, 35]. The PCR program included an initial denaturation (96 °C, 5 min), followed by 30 cycles (94 °C for 60 s, 78 °C PNA step for 5 s, 54 °C for 1 min, 74 °C for 60 s), followed by 74 °C for 10 min and then cooled down to 10 °C.

For the library preparation of the fungal community we used the primer pair ITS1f (5’- CTTGGTCATTTAGAGGAAGTAA-3’) and ITS2r (5’- GCTGCGTTCTTCATCGATGC-3’) [36, 37]. In the first PCR, 1 µL of DNA template was used for each 10 µL reaction; the reaction mixture contained 2 µL (5× Taq&Go), 1.2 µL (25 mM) MgCl2, 0.1 µL (10 µM) ITS1/ITS2 primers with pads, and 5.6 µL of PCR grade water. The second amplification was performed using 2 µL of the first PCR product in 30 µL reaction mixture. Each reaction mixture was composed of 6 µL (5x Taq&Go), 1.2 µL (10 µM) forward/reverse barcode primers, and 19.6 µL of PCR grade water. The PCR program for the first amplification step included an initial denaturation (96 °C, 5 min), followed by 35 cycles (95 °C for 30 s, 58 °C for 35 s, 72 °C for 40 s), followed by 72 °C for 10 min and then cool down to 10 °C. The subsequent reaction program involved: initial denaturation (95 °C, 5 min), then 15 cycles (95 °C for 30 s, 53 °C for 30 s, 72 °C for 30 s), followed by 5 min at 72 °C and cooling to 10 °C. PCR amplicons were purified using the Wizard SV Gel and PCR Clean-Up System (Promega, Madison, WI). Purified PCR amplicons were quantified using a Nanodrop 2000 (Thermo Scientific, Wilmington, DE, USA). Samples were combined in equimolar concentration and sequenced by Illumina MiSeq v2 (250 bp paired-end) amplicon sequencing. All raw reads obtained from the sequencing company were deposited at the NCBI under study accession number PRJNA1205945.

### Bioinformatics analysis

#### Sequence processing

Raw paired-end sequencing reads were subjected to quality control and demultiplexing using cutadapt v4.4 [38]. Demultiplexed reads were then processed within the open-source QIIME2 (version 2023.2.0) [39]. Primer sequences were removed, and quality filtering, denoising, and chimera removal were performed using the DADA2 algorithm [40] implemented in QIIME2. This process generated amplicon sequence variants (ASVs) and a corresponding feature table. Taxonomic assignments for bacterial sequences were made against the SILVA v138.1 reference database [41], while UNITE v10.5 [42] served as the reference for fungal sequences.

#### Statistical analysis

The biom files generated from sequencing was processed in R Version 4.4.0 in R Studio [43] for subsequent analysis. The decontam v1.22.0 package [44] was used to identify and remove potential contaminant taxa. The *isContaminant* function, utilizing the “prevalence” method, flagged taxa more prevalent in negative controls than true samples. Contaminant taxa, negative control samples, and any taxa or samples with zero abundance were removed using the *prune_taxa* and *prune_samples* functions from the phyloseq v1.46.0 [45]. For 16 S rRNA data, sequences identified as mitochondria, chloroplasts, archaea, and eukaryota were excluded using *subset_taxa* function. Similarly, ITS data were filtered by removing sequences classified as “NA” at the phylum level. All remaining taxa were pruned to ensure every sample retained at least one sequence. Rarefaction was performed at a minimum depth of 100 reads per sample for both bacterial and fungal communities using phyloseq. Alpha diversity was assessed using the Shannon index, calculated via the *estimate_richness* function [45]. To compare microbial diversity between moss and vascular plants, we conducted analysis of variance (ANOVA) followed by Tukey’s post hoc test. R² values from the ANOVA model were reported to assess the proportion of variance explained by host identity. Cumulative Sum Scaling (CSS) normalized data using *phyloseq_transform_css* function from metagMisc package v0.5.0 [46] to account for uneven sequencing depth. Differences in microbial community composition between mosses and vascular plants were analyzed using PERMANOVA *adonis2* function, from vegan package v2.6.6.1 [47] with 999 permutations. Community variation patterns were visualized through Principal Coordinate Analysis (PCoA) using the *ordinate* function from phyloseq. The p-values and R² values for Shannon diversity and community composition were obtained from the ANOVA and PERMANOVA model summaries, respectively. To assess the effect of soil type (carbonate vs. silicate soils) on microbial diversity and community composition, we selected a subset of 29 “twin pairs” (moss and vascular plant) out of the initial 52 pairs. This subset was chosen to ensure representation of both carbonate and silicate habitats. Specifically, the subset included 12 “twin pairs” from calcareous grasslands and 17 “twin pairs” from siliceous grasslands. Shannon diversity was calculated for this subset, and ANOVA was used to evaluate significant differences in diversity metrics between soil types. Community composition was analyzed using Bray-Curtis dissimilarity on CSS-transformed data, with PERMANOVA used to test for differences between soil types. Differential abundance of bacterial and fungal taxa between vascular plants and mosses was assessed using Linear Discriminant Analysis Effect Size (LEfSe) [48] implemented in the microbiomeMarker package v1.0.0 [49] in R. To identify biomarkers at the bacterial class level, a significance threshold of LDA score set as 1.0 and an adjusted p-value of 0.01. For the fungal analysis, a default threshold of LDA score set as 2.0 and an adjusted p-value of 0.01 were used to account for potential higher taxonomic diversity within fungal classes.

## Results

### Mosses exhibit higher bacterial but similar fungal diversity compared to vascular plants

**Fig. 1:**
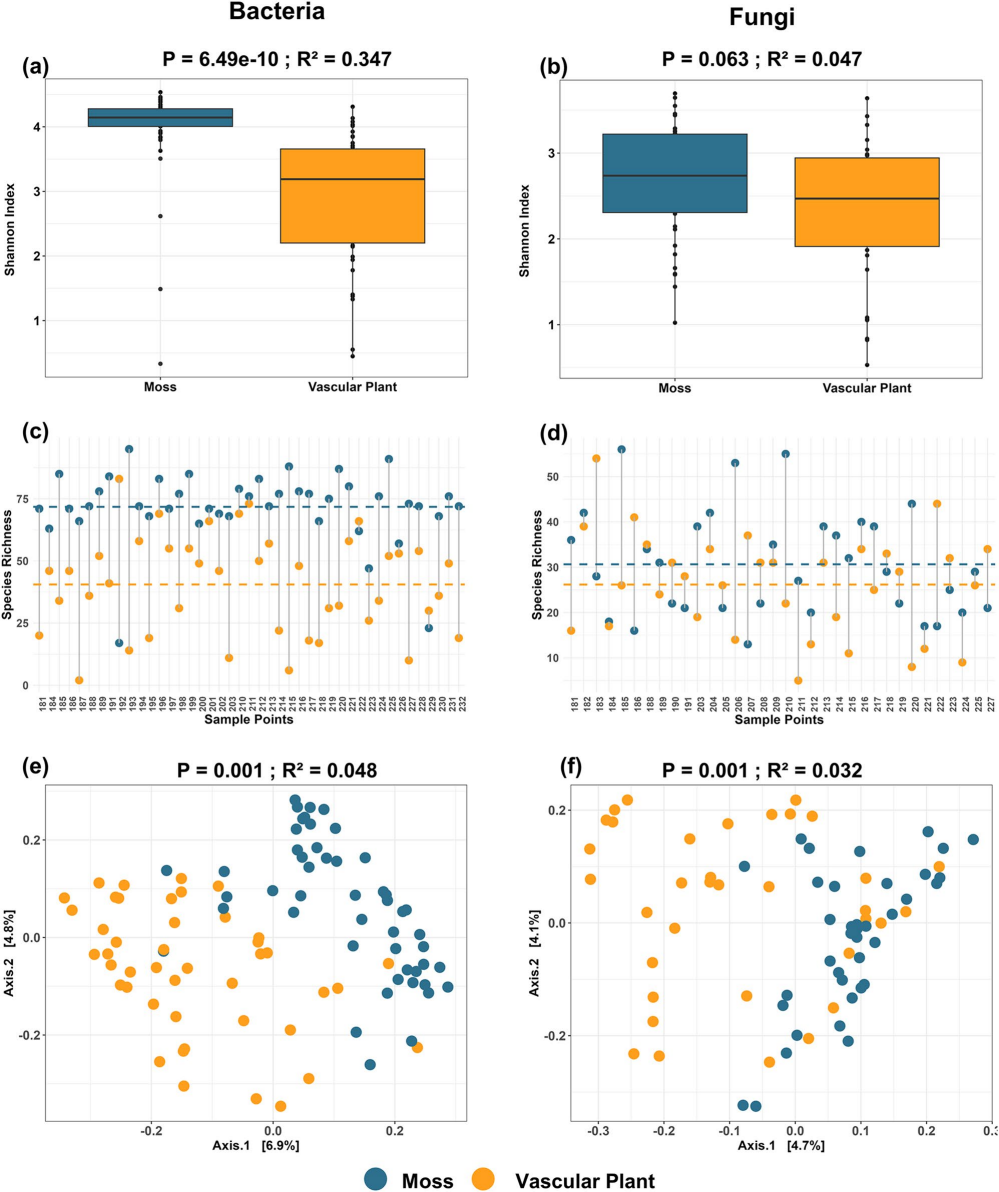
Phyllosphere microbial diversity and community composition differences between mosses and vascular plants. **(a)** Shannon diversity of bacterial communities, in mosses (blue) compared to vascular plants (orange) (ANOVA, *P* = 6.49e-10, R² = 0.347). **(b)** Shannon diversity of fungal communities, in mosses and vascular plants (ANOVA, *P* = 0.063, R² = 0.047). **(c)** Species richness (ASVs) of bacterial communities across individual samples. The horizontal dashed lines represent the mean richness for each group (Moss: dashed blue line, Vascular Plant: dashed orange line) **(d)** Species richness (ASVs) of fungal communities across individual samples. **(e)** Principal Coordinate Analysis (PCoA) of bacterial community composition based on Bray-Curtis dissimilarities (PERMANOVA, *P* = 0.001, R² = 0.048). **(f)** PCoA of fungal community composition based on Bray-Curtis dissimilarities, (PERMANOVA, *P* = 0.001, R² = 0.032)

In total, we recovered 3,435 bacterial and 1,174 fungal ASVs from mosses, and 1,760 bacterial and 911 fungal ASVs from vascular plants. Alpha diversity analysis (Shannon index) revealed a significantly higher bacterial diversity in mosses compared to vascular plants (*P* = 6.49e-10, R² = 0.347; Fig. 1a). In contrast, fungal Shannon diversity did not differ significantly between the two plant types (*p* = 0.063, R² = 0.047; Fig. 1b). On average, mosses harbored significantly higher bacterial species richness compared to vascular plants, with an average of 73 ASVs observed in mosses and 40 ASVs in vascular plants across all sampling points (Fig. 1c). For fungi, mosses exhibited a slightly higher average species richness compared to vascular plants, with 30 ASVs in mosses and 26 ASVs in vascular plants (Fig. 1d). However, this difference in fungal species richness was not statistically significant. Beta diversity analysis based on Bray-Curtis dissimilarity confirmed significant differences in microbial community composition between mosses and vascular plants for both bacterial (R² = 0.048, *P* = 0.001) and fungal (R² = 0.032, *P* = 0.001) communities (Fig. 1e and f). Although the first two PCoA axes explained a modest proportion of variance, the group separation was statistically supported by PERMANOVA, confirming the significant compositional differences between two plant types.

### Soil type shapes moss bacterial diversity and community composition

**Fig. 2:**
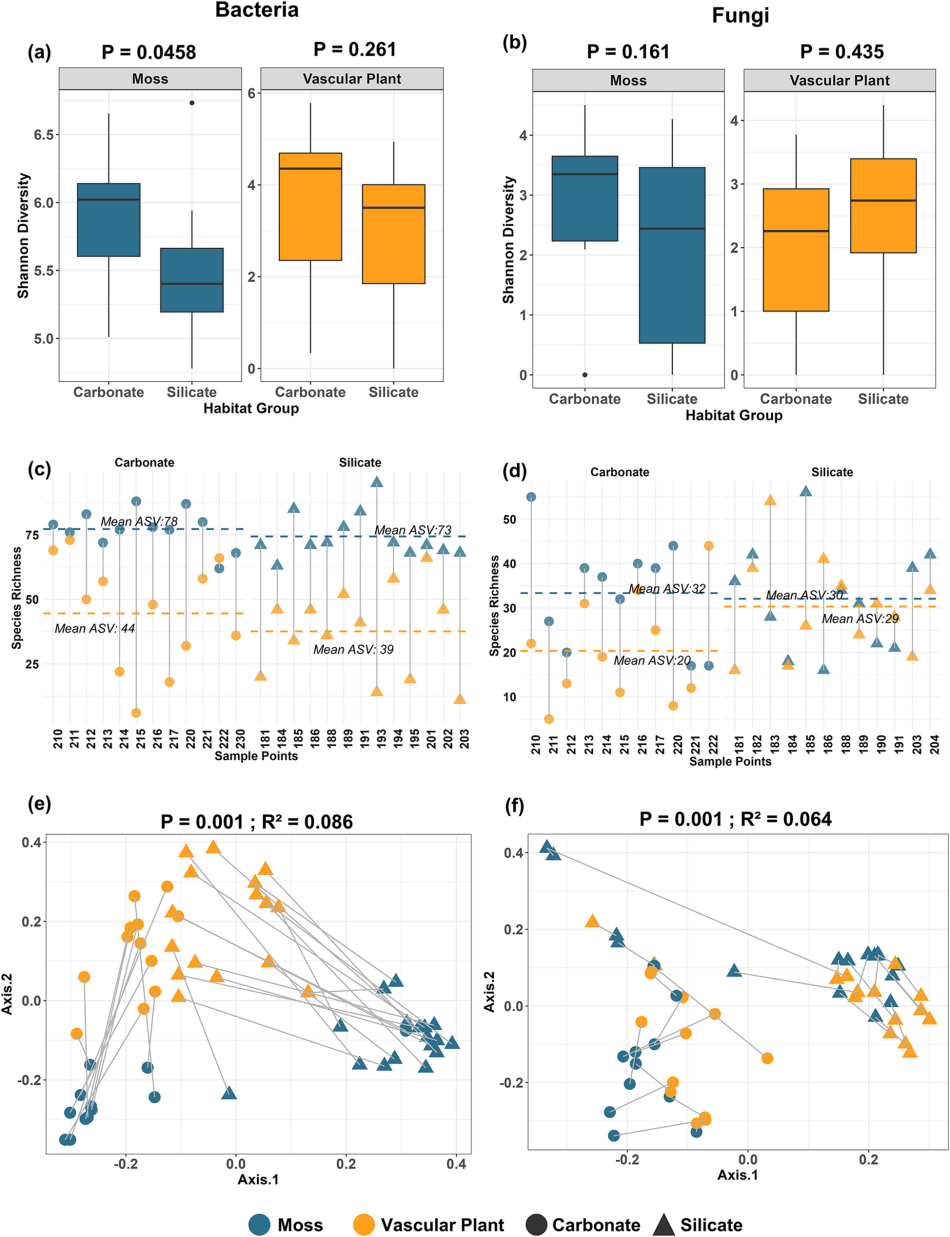
Influence of soil type (carbonate vs. silicate) on phyllosphere microbial diversity and community composition of mosses (blue) and vascular plants (orange). **(a)** Shannon diversity of bacterial communities in mosses and vascular plants across carbonate and silicate soils (ANOVA, *P* = 0.0458; *P* = 0.261). **(b)** Shannon diversity of fungal communities in mosses and vascular plants across soil types (ANOVA, *P* = 0.161; *P* = 0.435). **(c)** Species richness (ASVs) of bacterial communities across individual samples, separated by soil type. The horizontal dashed lines represent the mean richness for each group (Moss: dashed blue line, Vascular Plant: dashed orange line). **(d)** Species richness (ASVs) of fungal communities across individual samples, separated by soil type, with dashed lines representing the mean richness for each group. **(e)** Principal Coordinate Analysis (PCoA) of bacterial community composition based on Bray-Curtis dissimilarities by soil type for both plant types (PERMANOVA, *P* = 0.001, R² = 0.086). **(f)** Principal Coordinate Analysis (PCoA) of fungal community composition based on Bray-Curtis dissimilarities, by soil type (PERMANOVA, *P* = 0.001, R² = 0.064). Carbonate soils are indicated by circles and silicate soils by triangles

Shannon diversity index revealed a significant effect of soil type on bacterial diversity in mosses. Specifically, bacterial diversity was significantly higher in carbonate soils compared to silicate soils (*P* = 0.0458; Fig. 2a). However, for vascular plants, no significant difference in bacterial diversity was observed between soil types (*P* = 0.261; Fig. 2a). Fungal diversity did not exhibit significant differences between carbonate and silicate soils for either mosses (*P* = 0.161; Fig. 2b) or vascular plants (*P* = 0.435; Fig. 2b). Mosses consistently exhibited significantly higher bacterial species richness than vascular plants across both soil types. In carbonate soils, mosses hosted an average of 78 ASVs, nearly double the 44 ASVs observed in vascular plants across all sampling points (Fig. 2c). Similarly, in silicate soils, mosses demonstrated higher bacterial richness, with an average of 73 ASVs compared to 39 ASVs in vascular plants (Fig. 2c). For fungal communities, mosses also exhibited higher species richness in carbonate soils, with an average of 32 ASVs compared to 20 ASVs in vascular plants (Fig. 2d). However, in silicate soils, the fungal species richness between mosses and vascular plants was nearly equivalent, with mosses harboring an average of 30 ASVs and vascular plants hosting 29 ASVs across all sampling points (Fig. 2d). Bacterial community composition was significantly different between the two soil types for both mosses and vascular plants (R² = 0.086, *P* = 0.001; Fig. 2e). Similarly, the effect of soil type on fungal community composition was significant for both mosses and vascular plants (R² = 0.064, *P* = 0.001; Fig. 2f).

### Contrasting microbial enrichment patterns: mosses exhibit soil-type dependency while vascular plants show host-specific signatures

**Fig. 3:**
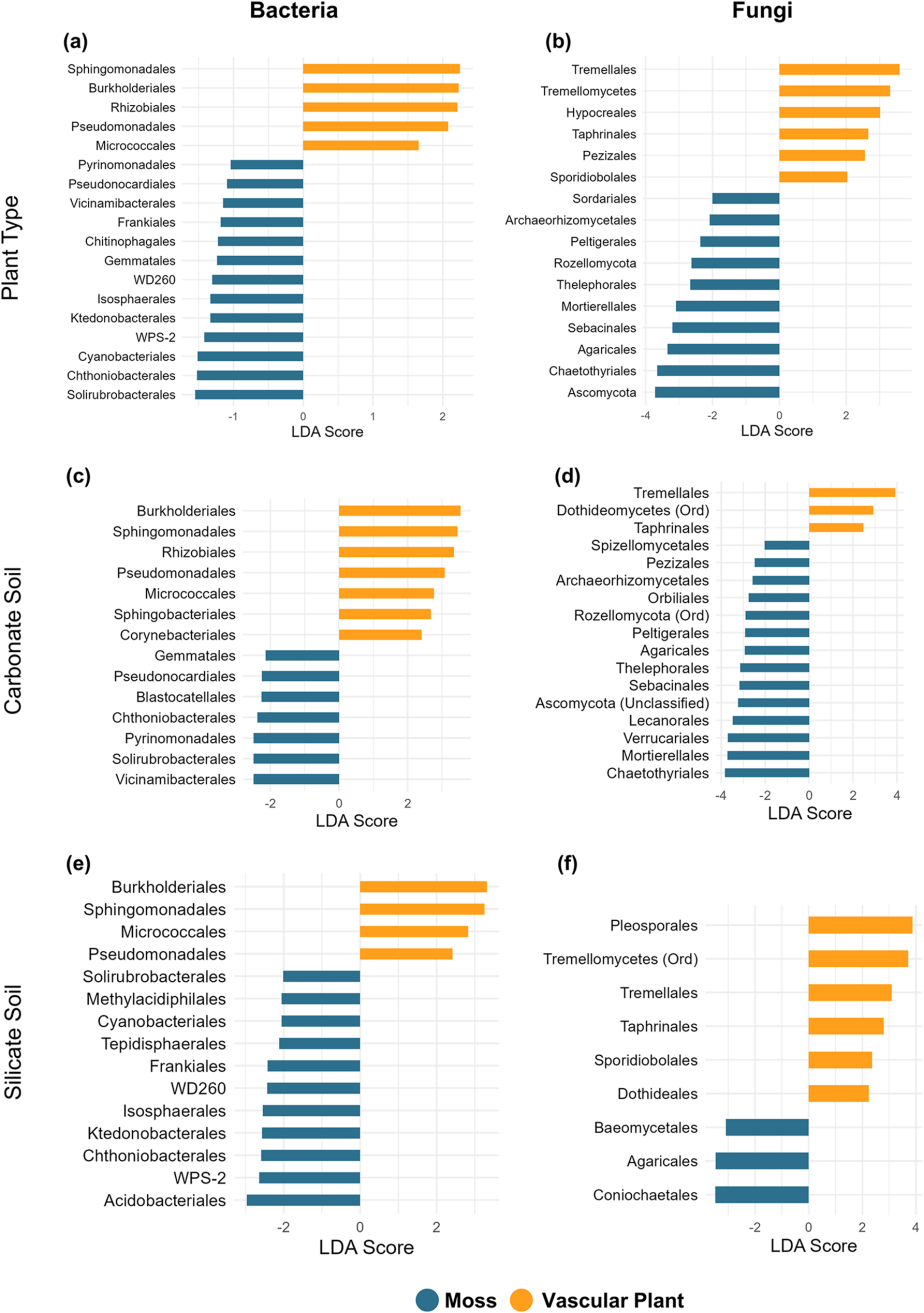
Differentially abundant bacterial and fungal orders in the phyllosphere of mosses (blue) and vascular plants (orange) based on Linear Discriminant Analysis Effect Size (LEfSe). **(a)** Bacterial orders enriched in mosses and vascular plants across all soil types. **(b)** Fungal orders enriched in mosses and vascular plants across all soil types. **(c)** Bacterial orders enriched in both mosses and vascular plants specifically in carbonate soils. **(d)** Fungal orders enriched in both mosses and vascular plants specifically in carbonate soils. **(e)** Bacterial orders enriched in both mosses and vascular plants specifically in silicate soils. **(f)** Fungal orders enriched in both mosses and vascular plants specifically in silicate soils. The length of the bar represents the effect size (LDA score)

To identify microbial enrichment patterns associated with plant types, we performed LEfSe analysis using the full dataset, which included all habitat types. To further explore the role of soil type, we conducted additional LEfSe analyses by subsetting the dataset into carbonate and silicate habitat groups. Across all habitats, vascular plants consistently showed enrichment in bacterial orders such as *Sphingomonadales*,* Rhizobiales*,* Burkholderiales*,* Pseudomonadales*, and *Micrococcales* (Fig. 3a). These patterns were consistent across carbonate and silicate soils in vascular plants, with no bacterial orders uniquely enriched in vascular plants within either of the soil type (Fig. 3c and e). For fungal communities, vascular plants showed enrichment in orders such as *Tremellales*,* Hypocreales*, and *Pezizales* (Fig. 3b). Among fungal orders such as *Tremellales* and *Taphrinales* were prominent across both soil types in vascular plants (Fig. 3b). In contrast, mosses demonstrated a more dynamic and environment dependent enrichment pattern. Bacterial orders such as *Cyanobacteriales*,* Ktedonobacterales*, and *Isosphaerales* were significantly enriched across all habitat types (Fig. 3a). When analyzed separately by soil type, distinct enrichment profiles emerged: in carbonate soils, mosses were enriched in bacterial orders like *Vicinimibacterales* and *Blastocatellales*, whereas silicate soils favored orders such as *Acidobacteriales*,* Methylacidiphilales*, and *Tepidisphaerales* (Fig. 3c and e). Fungal orders such as *Agaricales*,* Sebacinales*, and *Thelephorales* revealed significant enrichment across all habitat types (Fig. 3b). Further, soil type highlighted distinct patterns: in carbonate soils, mosses were enriched in fungal taxa such as *Mortierellales*, *Chaetothyriales*, and *Sebacinales*, whereas silicate soils favored unique enrichments in orders like *Coniochaetales* and *Baeomycetales* (Fig. 3d and f).

## Discussion

Our study revealed a significant difference in bacterial diversity between mosses and vascular plants, with mosses harboring a significantly higher diversity. Vascular plants are known to selectively recruit and interact with microbial communities through root exudates and other plant-derived compounds, which create nutrient rich microenvironments favoring copiotrophs [12, 13]. These exudates release degradable carbon and other resources that stimulate microbial activity and enable host specific selection [50, 51]. In contrast, mosses lack a selective host filtering ability due to their simple anatomical structure and the absence of true roots, which limits their capacity to actively recruit and modulate microbial communities via root exudates. Their ectohydric nature allows for water and nutrient uptake directly across the entire surface of the plant, bypassing specialized transport systems [11, 52–55]. Combined with their relatively undifferentiated tissue and minimal cuticular development, these traits likely reduce host-mediated microbial selection, which explains the influence of environmental factors on moss-associated microbial communities compared to vascular plants. Community composition analysis revealed distinct microbial communities associated with mosses and vascular plants, indicating host-specific microbial assembly. In mosses, we observed significant enrichment of bacterial orders including *Cyanobacteriales*,* Ktedonobacterales*,* Isosphaerales*, and *Solirubrobacterales*. The enrichment of *Cyanobacteria*, for instance, is consistent with their well-established ecological role in nitrogen fixation in moss associated environments, particularly how specific moss associated *Cyanobacteria* contribute to nitrogen input in nitrogen-limited habitats [56]. The enrichment of *Planctomycetes* orders such as *Gemmatales* and *Isosphaerales* in mosses likely due to their ability to degrade complex organic matter, including polyphenolic-rich moss cell walls. These bacteria have been shown to break down *Sphagnum* debris under fluctuating redox conditions, facilitating organic matter turnover in peatland ecosystems [57, 58]. Additionally, the presence of *Verrucomicrobia* suggests a role in methane cycling, similar to findings in peatland ecosystems [59, 60]. In vascular plants, we observed enrichment of *Proteobacteria*, particularly orders like *Sphingomonadales*,* Rhizobiales*,* Burkholderiales*, and *Pseudomonadales*. This aligns with previous findings [61] and likely reflects the presence of bacteria known for their association with plants [13, 50]. While alpha diversity of fungal communities did not significantly differ between mosses and vascular plants, our community composition analysis and LEfSe results revealed distinct fungal communities associated with each plant type. This suggests that host identity and niche specialization play a crucial role in shaping fungal community composition, even when overall diversity levels are similar. Mosses were enriched in *Ascomycota*, including *Agaricales*,* Sebacinales*, and *Thelephorales*, consistent with the dominance of this phylum in bryophyte-associated communities [52, 62–64]. The diverse array of enriched *Ascomycota* orders likely reflects their wide range of functional roles, from saprotrophy *(Archaeorhizomycetales)* to potential mycorrhizal associations (*Sebacinales)* [65] and phosphate solubilization (*Mortierellales*) [66, 67]. In contrast, vascular plants showed enrichment in *Tremellomycetes*, known for their lignin-degrading capabilities, may facilitate nutrient recycling by decomposing complex plant polymers [68]. This is particularly interesting considering that mosses lack lignin in their cell wall [69]. Orders like *Hypocreales* and *Pezizales* are potentially involved in beneficial interactions with their hosts. Their enrichment in vascular plants may reflect mutualistic interactions, such as ectomycorrhizal associations, which support nutrient exchange and enhance host plant resistance to pathogens [70, 71].

Our findings support the concept of the ‘calcareous riddle,’ where carbonate soils not only foster a greater diversity of plant species [25], but also contribute to increased microbial diversity in the plants. Specifically, bacterial diversity was significantly higher in carbonate soils compared to silicate soils for mosses. However, no such effect was observed in vascular plants. Moreover, fungal diversity did not show significant differences between the two soil types in either mosses or vascular plants. Community composition analysis revealed that both bacterial and fungal communities in mosses and vascular plants were significantly shaped by soil type. These findings align with previous research in alpine regions, where carbonate and silicate soil types were found to have no effect on fungal and bacterial diversity but significantly influenced community composition [26]. This consistency highlights that, while soil type may not always directly influence microbial diversity, it does act as a major driver of community composition across different plant types and habitats. The different responses observed in mosses and vascular plants suggest that host plant is potentially modulating through its physiological traits. Mosses are known to chemically interact with their environment by releasing species-specific compounds like polyphenols, which can influence microbial recruitment [72]. These interactions could partly explain the higher sensitivity of moss microbial communities to soil type compared to vascular plants. Another factor is the impact of calcium concentrations prevalent in carbonate soils. High calcium levels in carbonate soils may indirectly affect bryophyte microbial communities by altering nutrient availability and pH conditions [73, 74]. High calcium also can reduce moss growth by promoting phosphorus deficiency, as observed in calci-tolerant species [75], which supports a distinct microbial community in the moss. However, it is important to consider the increased bacterial diversity in mosses from carbonate soils is partially attributable to higher moss species richness. This observation aligns with the concept of the ‘calcareous riddle,’ which predicts higher biodiversity in carbonate environments. Future studies should explicitly investigate the relationship between moss species richness and bacterial diversity in these contrasting soil types.

Soil type exerted a strong influence on microbial community composition, as revealed by the distinct enrichment patterns in the mosses and vascular plants observed in carbonate and silicate soils. *Micrococcales*, enriched in vascular plants from carbonate soils, align with the findings of Wang et al. (2024) and Borsodi et al. (2021), which attribute their abundance to the high pH and calcium-rich conditions of alkaline soils [76, 77]. Mosses in carbonate soils, however, exhibited a unique fungal enrichment profile. In mosses, *Chaetothyriales* and *Sebacinales* were particularly prominent in carbonate soils. The enrichment of *Sebacinales* in mosses from carbonate soils likely reflects their stress adaptive traits and functional versatility. As a ubiquitous root endophyte known to establish mutualistic interactions across diverse plant hosts, including bryophytes, *Sebacinales* support nutrient transport and resilience [65]. Their increased abundance in calcareous, alkaline, and phosphorus limited soils has been linked to environmental filtering and beneficial roles in plant survival under edaphic stress [78–80]. The melanization of *Chaetothyriales*, as noted by Quan et al. (2020) and Muggia et al. (2021), likely provides stress tolerance against alkaline conditions and nutrient availability challenges, suggesting a long-standing ecological adaptation [81, 82]. Similarly, the presence of *Mortierellales*, known for their phosphorus-solubilizing abilities [83], suggests a crucial role in enhancing phosphorus availability, which is often limited in alkaline soils. This could contribute to moss growth and adaptation to carbonate-rich conditions. For mosses, *Acidobacteriales* and *Methylacidiphilales* were enriched in silicate soils, consistent with their known preference for acidic environments [84, 85]. This suggests an active role in organic matter decomposition and methane cycling, crucial processes in nutrient-limited acidic soils. Although, methane availability would need to be confirmed. The presence of *Coniochaetales* in mosses from silicate soils aligns with their acidophilic tendencies, further emphasizing the selective pressures exerted by soil pH [86]. In addition to soil chemistry, soil water content may influence microbial diversity, particularly in mosses due to their ectohydric nature [53, 55, 87]. However, soil moisture was not measured in this study, future research should include this parameter, as previous studies demonstrated that soil moisture significantly alters microbial community composition in bryophytes [88, 89]. Our findings highlight the remarkable microbial biodiversity associated with both mosses and vascular plants in alpine ecosystem. This microbial richness adds significantly to the known biodiversity of the region, especially when compared to the 34 macro-fungi determined during sampling [30]. In conclusion, we have identified a rich microbial biodiversity, particularly in mosses, that should be considered in biodiversity strategies. We must also point out that the phyllosphere is generally considered to be species-poor in comparison to the other plant compartments [3, 90].

Our research reveals the influence of plant type and soil type in shaping phyllosphere microbial communities through different ecological mechanisms in mosses and vascular plants. Vascular plants exhibited consistent microbial enrichments across soil types, indicative of strong host-driven selection, while mosses responded more dynamically to soil conditions, reflecting their structural simplicity and physiological permeability. This divergence in microbial assembly underscores the contrasting roles of deterministic and environmental processes across plant types. Notably, the sensitivity of moss associated microbiomes to soil chemistry also suggest their potential role as bioindicators of environmental change in alpine ecosystems. Future research should focus on determining the functional roles of these distinct microbial communities and their responses to environmental changes in these sensitive alpine ecosystems. Although, our study clearly demonstrates that host identity and soil type influence microbial communities, we acknowledge that future studies applying null model approaches, such as the β-nearest taxon index (βNTI) or Raup-Crick metrics [91, 92], could provide deeper understanding of the relative contributions of stochastic and deterministic processes shaping these communities.

## Conclusion

This study advances our understanding of the ecological processes shaping plant-microbe interactions by demonstrating the varying influences of both host traits and soil type on microbial community assembly in alpine ecosystems. These findings underscore the importance of considering both host traits and environmental factors to fully understand the drivers of microbial diversity and composition, providing new insights into the plant microbiome assembly and diversity. Altogether, we discovered a high microbial diversity associated with the different plants and mosses studied. In the phyllosphere of each plant and moss, around 100 different microorganisms were found, which greatly increases the local and mostly invisible biodiversity. Microbial diversity was influenced by the soil type as well, which means that both factors, plant genotype and soil type, have to be considered in studies assessing microbial diversity. Microbial diversity, which is crucial for One Health, should be implemented in biodiversity strategies and considered in long-term plans to protect nature and reverse the degradation of ecosystems.

## Electronic supplementary material

Below is the link to the electronic supplementary material.

Supplementary Material 1: Supplementary Table 1 provides detailed sampling information including twin pair id, sampling coordinates, habitat type, host plant identity (vascular plant or moss), and taxonomic classification.

## Data Availability

The datasets supporting the conclusions of this article are available in the Sequence Read Archive of NCBI. The raw sequence data 16 S rRNA gene and ITS region are available under the BioProject ID PRJNA1205945 https://www.ncbi.nlm.nih.gov/bioproject/PRJNA1205945.
